# Immunomodulatory effects of *Potentilla indica* and *Dendrophthoe pentandra* on mice splenocytes and thymocytes

**DOI:** 10.3892/etm.2014.1657

**Published:** 2014-04-02

**Authors:** HUI YING ANG, TAMILSELVAN SUBRAMANI, SWEE KEONG YEAP, ABDUL RAHMAN OMAR, WAN YANG HO, MOHD PUAD ABDULLAH, NOORJAHAN BANU ALITHEEN

**Affiliations:** 1 Department of Cell and Molecular Biology, Faculty of Biotechnology and Biomolecular Sciences, Universiti Putra Malaysia, Malaysia; 2 Institute of Bioscience, Universiti Putra Malaysia, Serdang, Selangor 43400, Malaysia; 3 The University of Nottingham Malaysia Campus, Semenyih, Selangor 43500, Malaysia

**Keywords:** immunomodulatory, Rosaceae, mistletoe, bromodeoxyuridine assay, MTT, spleen, thymus

## Abstract

Immunomodulators are agents that are able to stimulate or inhibit the immune response. The leaf extracts from *Potentilla indica* and *Dendrophthoe pentandra* were analyzed *in vitro* for immunomodulatory activity and an MTT colorimetric assay was conducted to determine the proliferation of mice splenocytes and thymocytes. A bromodeoxyuridine assay was performed to analyze DNA synthesis and the Trypan blue exclusion method was conducted to evaluate the changes in total cell population. The results indicated that treatment with *P. indica* and *D. pentandra* produced a time- and dose-dependent increase in cell viability and proliferation. Following 72 h of treatment with *P. indica* and *D. pentandra*, thymocyte proliferation was augmented by 18 and 41%, respectively and splenocyte proliferation increased by 35 and 42%, respectively, when compared with untreated cells. The present study demonstrated that these extracts may act as potential immunostimulants and, thus, represent an alternative source of immunomodulatory compounds for the treatment of human immune-mediated diseases.

## Introduction

A promising and recently identified alternative to classical antibiotic treatment is the use of immunomodulators, which enhance immune reactions via the stimulation of non-specific systems, such as granulocytes, macrophages, complement, certain T-lymphocytes and various effector substances (1,2). Several studies have focused on identifying compounds that are able to modulate the biological response of immune cells thereby enhancing the immunity of the host against various diseases (3,4). The increasing interest in folk medicine is due numerous well known plant remedies that are able to exert their anti-infective influence by directly affecting the pathogen, in addition to affecting immune cells by improving their activity. These effects were partially contributed to by the stimulation of the natural and adaptive defense mechanisms of the host organism (5). Recent advances have successfully identified a large number of macromolecules that regulate the host-defense system and the majority of which result in enhancement, amplification and/or diversion of immune responses in positive and therapeutically desirable directions (6).

*Potentilla indica* (formally known as *Duchesnea indica*) is a member of the Rosaceae family, which is native to eastern and southern Asia and is commonly termed a mock strawberry. It exhibits a moderate cytotoxic effect against various cancerous cell lines (7–10) and limited inhibitory activity against normal cell growth (11). However, few studies were conducted to investigate the additional bioactivities of this plant, such as its immunomodulatory effect. Conversely, *Dendrophthoe pentandra* is a type of mistletoe that grows on the rambutan tree. Although mistletoe is a parasitic plant, it has been widely administered in traditional medicine for the treatment of coughs and cancer, and as a diuretic agent. Furthermore, various antioxidant compounds, such as flavonol glycoside and quercitrin, have been isolated from the ethanol extract of *D. pentandra* and the compounds were shown to contribute to its high antioxidant activity (12). Moreover, it has been demonstrated to enhance the phagocytic activity of macrophages (9). To the best of our knowledge, there is currently no *in vitro* study on the immunoproliferative effect of the ethanol extract of *P. indica* and *D. pentandra* against splenocytes and thymocytes. Therefore, the aim of the present study was to determine the immunomodulatory effects of these plant extracts on mice splenocyte and thymocyte proliferation and viability.

## Materials and methods

### Plant materials

*P. indica* and *D. pentandra* were collected from George Town, Penang, Malaysia and were identified by the Forest Research Institute (Kuala Lumpur, Malaysia). The plant leaves were air-dried at room temperature and 2 g was ground, and soaked in 100 ml ethanol for three days. The extracts were filtered using Whatman filter paper grade 1 (Sigma-Aldrich, St. Louis, MO, USA) and dried via evaporation in a reduced-pressure atmosphere using an Aspirator A-3S (EYELA, Tohoku, Japan) at <45°C. This process was repeated three times and the yield was 8.7%, w/w. The dried residue was suspended in dimethylsulfoxide (DMSO; Fisher Scientific, Loughborough, UK) as extract stock. Briefly, 0.1 g dried extract was dissolved in 1 ml DMSO to prepare a 10 mg/ml stock extract. The sub-stock solution (0.2 mg/ml) was prepared by diluting 20 μl stock solution in 980 μl serum-free Dulbecco’s modified Eagle’s medium (DMEM; Sigma-Aldrich). The percentage of DMSO used was <0.5% and the stock and sub-stock solutions were stored at 4°C.

### Animals

Imprinting control region mice (age, 5–8 weeks) were used in the present study. The mice were purchased from the Animal House, Universiti Putra Malaysia (Selangor, Malaysia). The mice were housed under standard conditions at 25±2°C and fed with standard pellets and tap water. The mice were protected from stress. The present study was approved by the Institutional Animal Care and Use Committee of the Universiti Putra Malaysia (Ref: UPM/FPV/PS/3.2.1.551/AUP-R2).

### Preparation of mouse thymus and spleen cell suspensions

Following the sacrifice of the mice by cervical dislocation, the thymus and spleen were removed and washed three times using Hanks’ balanced salt solution (Sigma-Aldrich). The thymus and spleen were pulverized separately using a rubber syringe plunger and pushed through an 80 μm sterile wire mesh screen in phosphate-buffered saline (PBS), which was supplemented with 0.1% (w/v) bovine serum albumin (BSA) and 2 mg/ml EDTA (PBS/BSA/EDTA; Sigma-Aldrich) solution, to obtain a single cell suspension. For the spleen cell suspension, the red blood cells were removed using a lysis buffer and the spleen and thymus cell suspensions were washed twice using the PBS/BSA/EDTA solution and suspended in DMEM, which was supplemented with 10% heat inactivated fetal bovine serum (PAA, Pasching, Austria). Cell counting was performed using a hemocytometer to determine the number of lymphocytes within the cell suspension.

### MTT cell proliferation assay

The lymphocytes were harvested during the logarithmic growth phase and seeded in 96-well plates at a density of 5×10^5^ cells/ml with a final volume of 100 μl/well. Following incubation for 24 h, 100 μl *P. indica* or *D. pentandra* extract (200 μg/ml) was loaded into the well plates and serially diluted. After 24, 48 and 72 h treatment, 20μl MTT (5 mg/ml) was added to each well for 4 h. Subsequently, the supernatant was removed and the MTT crystals were solubilized with 100 μl anhydrous DMSO per well. Thereafter, the cell viability was measured using a μQuant™ ELISA reader (BioTek Instruments Inc., Winooski, VT, USA) at 570 nm absorbance and the percentage of cell proliferation was calculated.

### Bromodeoxyuridine (BrdU) incorporation assay

A 96-well plate was used to allocate the different concentrations of extracts (100, 50 and 1 μg/ml) from either *P. indica* or *D. pentandra* for incubation with the splenocytes at 5×10^5^ cells/ml media. A BrdU ELISA kit (Chemicon, Temecula, CA, USA) was used with splenocytes, according to the manufacturer’s instructions, to estimate the extent of cell proliferation. The three incubation periods (24, 48 and 72 h) per extract were analyzed three times and three independent repeats of the assay were performed on the cells from the control group. The plate was read at an absorbance of 450 nm using the μQuant ELISA reader.

### Trypan blue exclusion method

The splenocytes and thymocytes (5×10^5^ cell/ml) were treated with 100 or 50 μg/ml *P. indica* or *D. pentandra* extract in 6-well plates for either 24, 48 or 72 h. The untreated cells served as the negative control. Following the incubation period, the cells were harvested and pelleted at 200 × g for 10 min. The pellets were suspended in 0.4% Trypan blue dye (Sigma-Aldrich) and 10 μl mixture was placed in a hemocytometer (Sigma-Aldrich) and the cells were counted under a phase contrast light microscope (Eclipse Ti, Nikon, Melville, NY, USA). Each of the extracts and the control were assayed five times in triplicate.

### Statistical analysis

The results were expressed as the mean ± standard error of the mean and statistical analyses were performed using SPSS version 16.0 (SPPS Inc., Chicago, IL, USA). The differences between the means were evaluated using one way analysis of variance, followed by Duncan’s test and P≤0.05 was considered to indicate a statistically significant difference.

## Results and Discussion

### Effects on splenocyte and thymocyte proliferation observed by MTT assay

**Figure 1:**
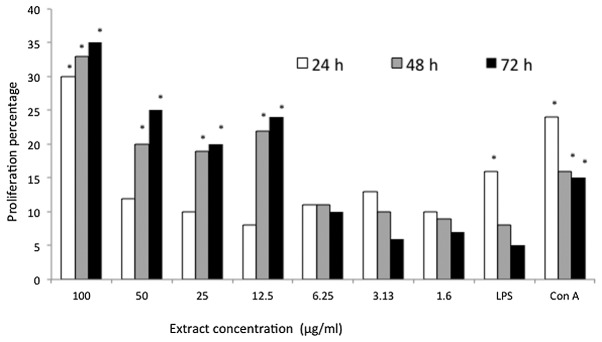
Proliferation effect of *Potentilla indica* on mice splenocytes at different concentrations (100, 50, 25, 12.5, 6.25, 3.13, 1.6 μg/ml and vehicle treated) for different incubation periods (24, 48 and 72 h) determined via MTT assay. Each value represents the mean ± standard error of the mean for three assays conducted three times. The differences between the control and treatment group were determined using one-way analysis of variance. ^*^P≤0.05 indicated a statistically significant difference vs. vehicle treated cells. LPS, lipopolysaccharide; Con A, concanavalin A.

**Figure 2:**
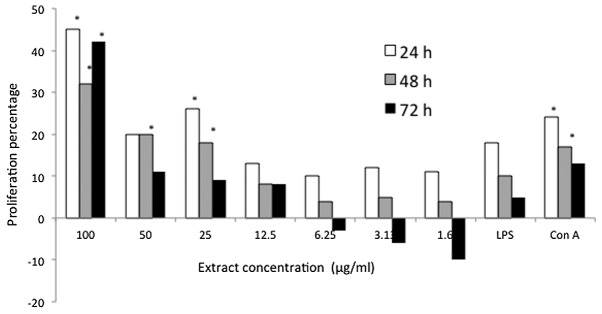
Proliferation effect of *Dendrophthoe pentandra* on mice splenocytes at different concentrations (100, 50, 25, 12.5, 6.25, 3.13, 1.6 μg/ml and vehicle treated) for different incubation periods (24, 48 and 72 h) determined via MTT assay. Each value represents the mean ± standard error of the mean for three assays conducted three times. The differences between the control and treatment group were determined using one-way analysis of variance. ^*^P≤0.05 indicated a statistically significant difference vs. vehicle treated cells. LPS, lipopolysaccharide; Con A, concanavalin A.

The predominant function of the thymus is to develop immature T cells into immunocompetent T cells, thus, the thymus contains 99% of mature T lymphocytes (13). By contrast, the spleen contains a relatively homogenous fraction of B and T lymphocytes, consisting of ~60% B cells and 40% T cells (14). Thus, an evaluation of the immunoproliferative effect on these cells provides an understanding of the influence of the leaf extract on T and B cells. The MTT-based lymphocyte proliferation assay was performed on specific immune cells at different incubation periods and the proliferation effects of *P. indica* and *D. pentandra* were analyzed using various stock concentrations, between 1.563 and 100 μg/ml. Two mitogens were used in the present study; lipopolysaccharide (LPS) as the B cell mitogen and concanavalin A (Con A) as the T cell mitogen. All of the *P. indica* extract concentrations stimulated the proliferation of the mice splenocytes at all of the incubation times; however, an inhibition of 0.1256% was observed at the 6.25 μg/ml concentration following 48 h of incubation (Fig. 1). By contrast, the proliferation of the mice splenocytes induced by 100 μg/ml *P. indica* extract increased from 30% at 24 h to 33% after 48 h of treatment. The greatest proliferation (35%) was obtained following 72 h of treatment with the same concentration of extract. Furthermore, the two positive controls were observed to stimulate proliferation of the mice splenocytes in a time-dependent manner. However, treatment with *P. indica* extract indicated an improved stimulatory effect, exhibiting 30 and 19% more proliferation than the LPS and Con A groups, respectively, following 72 h of treatment. Furthermore, the predominant immunostimulatory effect of *D. pentandra* extract on the mice splenocytes was observed at a concentration of 100 μg/ml (Fig. 2). The highest proliferation was observed in the mice splenocytes that were treated with 100 μg/ml *D. pentandra* extract, although the immunostimulatory effect of *D. pentandra* extract on the mice splenocytes was markedly weaker at low concentrations. Overall, the pattern of mouse splenocyte proliferation, induced by *P. indica* and *D. pentandra* extracts, were somewhat comparable as the two extracts exhibited the majority of active proliferation at a concentration of100 μg/ml.

**Figure 3:**
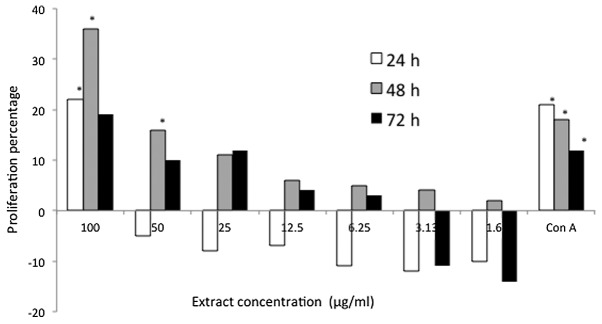
Proliferation effect of *Potentilla indica* on mice thymocytes at different concentrations (100, 50, 25, 12.5, 6.25, 3.13, 1.6 μg/ml and vehicle treated) for different incubation periods (24, 48 and 72 h) determined via MTT assay. Each value represents the mean ± standard error of the mean for three assays conducted three times. The differences between the control and treatment group were determined using one-way analysis of variance. ^*^P≤0.05 indicated a statistically significant difference vs. vehicle treated cells. Con A, concanavalin A; LPS, lipopolysaccharide.

**Figure 4:**
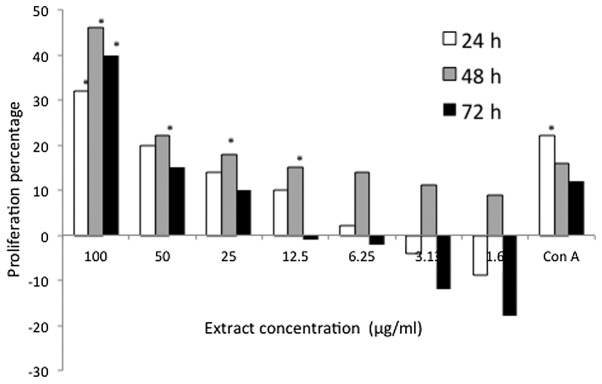
Proliferation effect of *Dendrophthoe pentandra* on mice thymocytes at different concentrations (100, 50, 25, 12.5, 6.25, 3.13, 1.6 μg/ml and vehicle treated) for different incubation periods (24, 48 and 72 h) determined via MTT assay. Each value represents the mean ± standard error of the mean for three assays conducted three times. The differences between the control and treatment group were determined using one-way analysis of variance. ^*^P≤0.05 indicated a statistically significant difference vs. vehicle treated cells. Con A, concanavalin A; LPS, lipopolysaccharide.

In addition, P. *indica* exhibited a marked proliferation effect on T cells, exhibiting induction of 21, 35 and 17% after treatment for 24, 48 and 72 h, respectively, at a concentration of 100 μg/ml (Fig. 3). The highest stimulatory effect by *P. indica* extract on the mouse thymocytes (proliferation, 35%) was observed following 48 h at a concentration of 100 μg/ml. Moreover, it was evident that the proliferation of mouse thymocytes reduced significantly throughout the treatment periods when they were treated with low concentrations (≤50 μg/ml) of *P. indica* extract. When compared with the stimulatory effect of 100 μg/ml *P. indica* extract, thymocyte proliferation, which was induced by the extract remained higher than the proliferation that was induced by Con A. A notable proliferation effect was observed at the highest concentration (100 μg/ml) of *D. pentandra* extract, with values of 33, 44 and 41% observed throughout the 24, 48 and 72 h treatment periods, respectively (Fig. 4). Similarly, the stimulatory effect of 100 μg/ml *D. pentandra* extract on mouse thymocyte proliferation was greater than that of the positive control.

### BrdU incorporation assay on splenocytes

**Figure 5:**
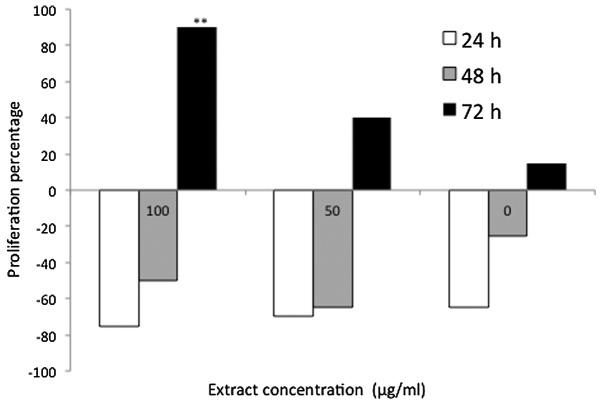
Bromodeoxyuridine proliferation effect of *Potentilla indica* extract on mice splenocytes at different concentrations [0 (vehicle treated), 50 and 100 μg/ml] for different incubation periods (24, 48 and 72 h). Each value represents the mean ± standard error of the mean for three assays conducted three times. The differences between the control and treatment group were determined using one-way analysis of variance. ^*^P≤0.05 indicated a statistically significant difference vs. vehicle treated cells.

**Figure 6:**
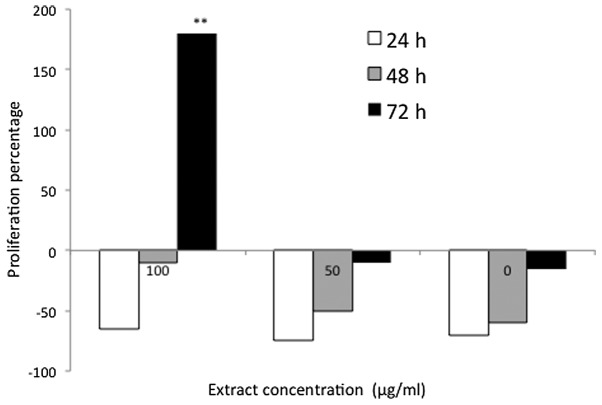
Bromodeoxyuridine proliferation effect of *Dendrophthoe pentandra* extract on mice splenocytes at different concentrations [0 (vehicle treated), 50 and 100 μg/ml] for different incubation periods (24, 48 and 72 h). Each value represents the mean ± standard error of the mean for three assays conducted three times. The differences between the control and treatment group were determined using one-way analysis of variance. ^*^P≤0.05 indicated a statistically significant difference vs. vehicle treated cells.

DNA synthesis in cells treated with *P. indica* extract for 24 and 48 h was markedly reduced at all of the concentrations that were employed in the present study (100, 50 and 1 μg/ml), indicating a non-stimulatory effect, which ranged from −28 to −78% (Fig. 5). An incremental increase in DNA synthesis in the *P. indica*-treated cells was observed following a prolonged incubation period of 72 h with 1, 50 and 100 μg/ml *P. indica* extract; the DNA synthesis rate was 87, 18 and 42%, respectively. The *D. pentandra*-treated cells exhibited the greatest rate of DNA synthesis, which reached 187% following 72 h of treatment with 100 μg/ml *D. pentandra* extract (Fig. 6). This demonstrated a 100% elevation in the proliferation rate of the *D. pentandra*-treated cells when compared with the *P. indica*-treated cells under matching concentration and incubation conditions. However, this was the only positive result that was observed in the *D. pentandra*-treated cells, whereas the non-stimulatory effect, ranging from −9 to −81%, was observed at all of the other concentrations and incubation periods. These results indicated that *P. indica*- and *D. pentandra*-treated cells exhibited the highest rate of DNA synthesis following 72 h of treatment, when the greatest concentration (100 μg/ml) was used. The cells were sustained for longer in the medium of the two extracts without exhibiting signs of inhibition or toxicity, however, MTT assays have previously been reported to overestimate proliferation (15) or underestimate the growth inhibitory effects of specific cytokines (16). These assays exhibited less sensitivity when compared with fluorescent labeling techniques (17) and occasionally failed to detect the proliferation of lymphocytes (18–20). In the present study, as the MTT and BrdU assays indicated positive correlations, it could be deduced that these techniques were effective in detecting the proliferation of mice splenocytes and thymocytes.

### Trypan blue exclusion assay

**Figure 7:**
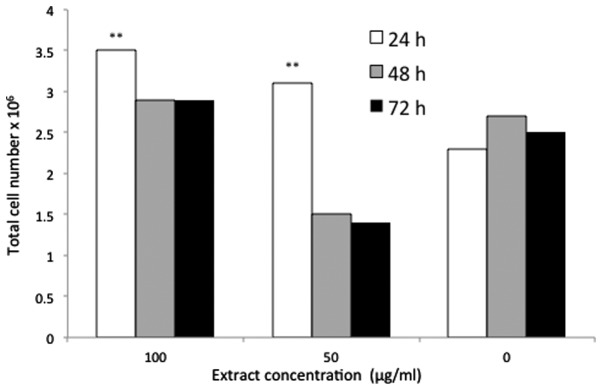
Effect of *Potentilla indica* on trypan blue cell viability count of mice splenocytes at different concentrations [0 (vehicle treated), 50 and 100 μg/ml] after 24, 48 and 72 h of treatment. Each value represents the mean ± standard error of the mean for three assays conducted three times. The differences between the control and treatment group were determined using one-way analysis of variance. ^*^P≤0.05 indicated a statistically significant difference vs. vehicle treated cells.

**Figure 8:**
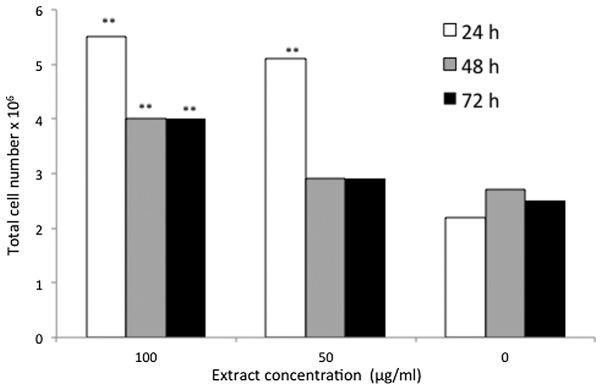
Effect of *Dendrophthoe pentandra* on trypan blue cell viability count of mice splenocytes at different concentrations [0 (vehicle treated), 50 and 100 μg/ml] after 24, 48 and 72 h of treatment. Each value represents the mean ± standard error of the mean for three assays conducted three times. The differences between the control and treatment group were determined using one-way analysis of variance. ^*^P≤0.05 indicated a statistically significant difference vs. vehicle treated cells.

The total cell population was increased to 3.5×10^6^, 2.85×10^6^ and 2.8×10^6^ cells following treatment for 24, 48 and 72 h, respectively, with 100 μg/ml *P. indica* extract. The total cell number (3.5×10^6^) was the highest proliferation rate achieved as a result of treatment with *P. indica* extract, which was followed by 3.1×10^6^ cells that was observed following 24 h of treatment with 50 μg/ml *P. indica* extract (Fig. 7). The greatest cell number (5.54×10^6^ cells) was achieved following 24 h of treatment with 100 μg/ml *D. pentandra*, this was followed by 5.12×10^6^ cells at the concentration of 50 μg/ml. Following 48 and 72 h, the total cell population resulting from treatment with 100 μg/ml *D. pentandra* extract did not differ; 4.16×10^6^ cells were obtained over the two time periods. Moreover, a comparable pattern was observed in the *D. pentandra* treatment group at a concentration of 50 μg/ml, where 2.88×10^6^ cells were obtained following incubation for 48 and 72 h (Fig. 8). The two extracts demonstrated that increases in cell number were time- and dose-dependent and the highest cell population count was observed following 24 h of incubation with the greatest concentration (100 μg/ml) of *P. indica* or *D. pentandra* extract. When comparing the two extracts, *D. pentandra* exhibited a greater stimulatory effect regarding the number of viable cells, the rate of DNA synthesis and the total cell population. However, the effect of these extracts requires further investigation to isolate and evaluate the active metabolites from the extracts, which contribute to the immunomodulatory effect on thymocytes and splenocytes.

In conclusion, *P. indica* and *D. pentandra* extracts stimulated the proliferation of mice splenocytes and thymocytes in a time- and dose-dependent manner. The *D. pentandra* extracts demonstrated a greater stimulatory effect on mice splenocytes and thymocytes when compared with the *P. indica* extracts. The ability of these plant extracts to modulate innate immune functions suggests promising further therapeutic development on wound healing and inhibition of tumor growth through modulation of lymphocytes.
